# A Second Medical School in Louisville

**Published:** 1849-02

**Authors:** 


### A SECOND MEDICAL SCHOOL IN LOUISVILLE.

We learn that a number of the physicians of Louisville have memorialized the Legislature of Kentucky, praying for a charter for a second Medical School in this city. What the fate of this petition may be we are not able to predict, but we feel quite safe in saying that if the prayer should be granted and another school attempted here, the effect will be injurious to the interests of medicine not only in Kentucky, but throughout the country. The multiplication of medical schools is beginning to be felt as one of the great evils by which the profession is afflicted. If the professional sentiment of Kentucky could be expressed upon the subject, we are very sure that it would be found to be in opposition to the project of a second school, and in favor of cherishing one institution in such a way as to render it an ornament to the State. We confess we look with great confidence to the wisdom of our Legislature, and shall be surprised if they adopt a policy so detrimental to the true interests of medical science in the Commonwealth. The experience of the cities around us has shown how hopeless is the endeavor to build up two schools of medicine amid a population as limited as that of Louisville. We do not believe this experience will be disregarded by the wise framers of our laws, however it may have been wasted upon the authors of this insane proposition.

